# Lack of Cdkl5 Disrupts the Organization of Excitatory and Inhibitory Synapses and Parvalbumin Interneurons in the Primary Visual Cortex

**DOI:** 10.3389/fncel.2016.00261

**Published:** 2016-11-28

**Authors:** Riccardo Pizzo, Antonia Gurgone, Enrico Castroflorio, Elena Amendola, Cornelius Gross, Marco Sassoè-Pognetto, Maurizio Giustetto

**Affiliations:** ^1^Department of Neuroscience, University of TurinTurin, Italy; ^2^Molecular Medicine and Medical Biotechnologies, University of Naples “Federico II”Napoli, Italy; ^3^Mouse Biology Unit, European Molecular Biology Laboratory (EMBL)Monterotondo, Italy; ^4^National Institute of Neuroscience-ItalyTurin, Italy

**Keywords:** Rett syndrome (RTT), CDKL5, cerbral cortex, synapses, mouse models

## Abstract

Cyclin-dependent kinase-like 5 (CDKL5) mutations are found in severe neurodevelopmental disorders, including the Hanefeld variant of Rett syndrome (RTT; CDKL5 disorder). CDKL5 loss-of-function murine models recapitulate pathological signs of the human disease, such as visual attention deficits and reduced visual acuity. Here we investigated the cellular and synaptic substrates of visual defects by studying the organization of the primary visual cortex (V1) of Cdkl5^−/y^ mice. We found a severe reduction of c-Fos expression in V1 of Cdkl5^−/y^ mutants, suggesting circuit hypoactivity. Glutamatergic presynaptic structures were increased, but postsynaptic PSD-95 and Homer were significantly downregulated in CDKL5 mutants. Interneurons expressing parvalbumin, but not other types of interneuron, had a higher density in mutant V1, and were hyperconnected with pyramidal neurons. Finally, the developmental trajectory of pavalbumin-containing cells was also affected in Cdkl5^−/y^ mice, as revealed by fainter appearance perineuronal nets at the closure of the critical period (CP). The present data reveal an overall disruption of V1 cellular and synaptic organization that may cause a shift in the excitation/inhibition balance likely to underlie the visual deficits characteristic of CDKL5 disorder. Moreover, ablation of CDKL5 is likely to tamper with the mechanisms underlying experience-dependent refinement of cortical circuits during the CP of development.

## Introduction

*De novo* mutations of the cyclin-dependent kinase-like 5 (*CDKL5*) gene are responsible for the Hanefeld variant of Rett syndrome (RTT), also referred to as CDKL5 disorder. This is an *X*-linked neurodevelopmental condition with a broad range of deficits including stereotypical hand movements, deficient language acquisition and in some cases respiratory dysregulation. Differently from classic RTT, patients affected by CDKL5 disorder exhibit early onset (in the first months of life) epilepsy, severe hypotonia, characteristic sideways glance, abnormal eye tracking and severe visual impairment (Mari et al., 2005; Bertani et al., 2006).

CDKL5 encodes a ubiquitously expressed serine/threonine kinase whose catalytic domain shares homology with members of the cyclin-dependent kinase family and mitogen-activated protein kinases (Montini et al., 1998). This kinase is expressed at high levels in the brain, reaching a peak during postnatal development, when crucial events occur, such as neuronal maturation and synaptogenesis (Rusconi et al., 2008; Kilstrup-Nielsen et al., 2012). CDKL5 can be found both in the nucleus and the cytoplasm, as it shuttles between these cellular compartments. Recently, it has been demonstrated that CDKL5 is targeted to synapses via its interaction with the palmitoylated form of postsynaptic density protein-95 (PSD-95; Zhu et al., 2013). Moreover, by phosphorylating netrin-G1 ligand (NGL-1), CDKL5 can modulate the association of this cell adhesion molecule with PSD-95, thereby contributing to regulate the structural organization of dendritic spines and excitatory synapse function (Ricciardi et al., 2012).

To clarify the role of CDKL5 in the etiology of CDKL5 disorder, we and others previously generated and characterized a constitutively Cdkl5 knockout (KO) mouse (Wang et al., 2012; Amendola et al., 2014). We showed that Cdkl5 KOs exhibit behavioral abnormalities that resemble RTT-like phenotypes, such as hind-limb clasping, hypoactivity and visual attention/acuity deficits (Amendola et al., 2014). These defects are associated with neuroanatomical alterations, such as a significant reduction of the thickness of somatosensory cortex and hippocampus, and a decreased length and complexity of dendritic arborization of pyramidal neurons (Amendola et al., 2014; Fuchs et al., 2015). Cdkl5-KO mice also present severe deficits in the organization and stability of dendritic spines, as well as in the density of PSD-95 dendritic clusters and synaptic long-term potentiation (Della Sala et al., 2016).

Despite these advances, a comprehensive understanding of the role of CDKL5 in the organization and function of cortical circuitry is still missing. Since RTT might arise from the disruption of the balance between excitation and inhibition (E/I) in specific brain circuits (Rubenstein and Merzenich, 2003; Boggio et al., 2010; Zoghbi and Bear, 2012; Feldman et al., 2016), we hypothesized that CDKL5 could have a key role in this process. In the present study, we evaluated whether structural E/I abnormalities could result from the lack of CDKL5 by investigating the organization of excitatory and inhibitory synapses in the cerebral cortex of Cdkl5 KO mice. Given that visual deficits are a key sign of CDKL5 disorder both in patients and in mouse models (Bahi-Buisson et al., 2008; Moseley et al., 2012; Amendola et al., 2014), we focused our attention on the primary visual cortex (V1) of adult mutant mice. The well-characterized developmental pattern of V1 circuitry (Levelt and Hübener, 2012) also allowed us to establish whether the absence of CDKL5 might interfere with the experience-dependent maturation processes during the critical period (CP) of V1 refinement.

## Materials and Methods

### Animals

Animal care and handling throughout the experimental procedures were conducted in accordance with European Community Council Directive 86/609/EEC for care and use of experimental animals with protocols approved by the Italian Minister for Scientific Research (Authorization number 175/2015-PR) and the Bioethics Committee of the University of Torino, Italy. Animal suffering was minimized, as was the number of animals used. Mice for testing were produced by crossing Cdkl5^−/x^ females with Cdkl5^−/y^ males and Cdkl5^−/x^ females with Cdkl5^+/y^ males (Amendola et al., 2014). Littermate controls were used for all the experiments. After weaning, mice were housed 3–5 per cage on a 12 h light/dark cycle (lights on at 7:00 h) in a temperature-controlled environment (21 ± 2°C) with food and water provided *ad libitum*. For most of this study, we used 8-week old Cdkl5^−/y^ males; for the evaluation of CP abnormalities, brains were collected from Cdkl5^−/y^ male mice aged 18, 22 or 35 days.

### Immunocytochemical and Histochemical Procedures

For most immunolabeling experiments, animals were anesthetized with an intraperitoneal injection of Avertin (Sigma-Aldrich) and transcardially perfused with ice-cold 4% formaldehyde in 0.1 M phosphate buffer (PB). The brains were then dissected and kept in the same fixative solution overnight at 4°C. Afterwards, brains were cryoprotected by immersion in 10%, 20%, and 30% sucrose solutions, cut into 30 μm sections with a cryostat and stored at −20°C in a solution containing 30% ethylene glycol and 25% glycerol until use. Cryosections were subsequently processed free-floating for immunohistochemistry or peri-neuronal nets (PNNs) visualization (Ricciardi et al., 2011; Tomassy et al., 2014).

For c-Fos detection, after a blocking step in phosphate-buffered saline (PBS) containing 10% normal goat serum (NGS) and 0.05% Triton X-100, sections were incubated overnight at room temperature with rabbit anti-c-Fos (1:500, Santa Cruz Biotechnology, Santa Cruz, CA, USA, cat. sc-52), diluted in PBS with 3% NGS and 0.05% Triton X-100. Sections were then rinsed, incubated for 1 h with goat anti-rabbit biotinylated secondary antibody (1:250; Vector Labs, Burlingame, CA, USA), and transferred to a biotin-avidin complex containing solution (1:100, Vector Labs, Burlingame, CA, USA). The immunoreactivity was visualized by incubating the sections in a solution containing 3,3^′^-diaminobenzidine (0.05% DAB in Tris-HCI, pH 7.6) with 0.01% H_2_O_2_ for 3 min. Sections were mounted on gelatin-coated glass slides, air dried, dehydrated in ethanol, cleared in xylene, mounted in DPX mounting media (Fisher Scientific, Loughborough, UK) and coverslipped. Control sections in which the primary antiserum was omitted were completely unstained (not shown). Sections were observed with a light microscope (Eclipse 800, Nikon, Tokyo, Japan) equipped with a CCD camera (Axiocam HRc, Zeiss, Jena, Germany). Images of V1 (from the pial surface to the white matter) were captured with a 10× objective from 4 to 6 animals per genotype using at least two corresponding coronal brain sections.

For immunofluorescence, sections were kept in PBS solution containing 0.05% Triton X-100 and 10% normal donkey serum (NDS) for 1 h followed by an overnight incubation at 25°C with the appropriate primary antibodies (see Table 1). Antibodies were diluted in PBS with 3% NDS and 0.05 Triton X-100. The following day the sections were washed and incubated with appropriate fluorescent secondary antibodies (1:1000; Jackson ImmunoResearch, West Grove, PA, USA). After several PBS rinses, the sections were mounted on gelatin-coated glass slides and coverslipped with Dako fluorescence mounting medium (Dako Italia, Milan, Italy).

**Table 1:** **Primary antibodies used in this study**.

Primary antibodies	Species of origin	Working dilution	Supplier and catalog no.
anti-CR	Guinea pig	1:500	Synaptic Systems, Göttingen, Germany, cat. 214104
anti-c-Fos	Rabbit	1:1500	Santa Cruz Biotechnology, Santa Cruz, CA, USA, cat. sc-52
anti-Gephyrin	Mouse	1:500	Synaptic Systems, Göttingen, Germany, cat. 147 011
anti-pan-HOMER	Rabbit	1:500	Synaptic Systems, Göttingen, Germany, cat. 160–103
anti-NeuN	Mouse	1:200	Millipore, Darmstadt, Germany, cat. MAB377
anti-PSD-95	Mouse	1:500	NeuroMab, CA, USA, cat. 75–028
anti-PV	Mouse	1:10.000	Swant, Marly, Switzerland, cat. 235
anti-PV	Guinea pig	1:500	Synaptic Systems, Göttingen, Germany, cat. 195004
anti-SOM	Rat	1:250	Millipore, Darmstadt, Germany, cat. MAB354
anti-VGAT	Rabbit	1:500	Synaptic Systems, Göttingen, Germany, cat. 131–002
anti-VGluT1	Guinea pig	1: 5000	Millipore, Darmstadt, Germany, cat. 5905
anti-VGluT2	Guinea pig	1: 2000	Millipore, Darmstadt, Germany, cat. 2251

To visualize PNNs, sections were incubated overnight in biotinylated Wisteria floribunda agglutinin (WFA; 1:200, Sigma Aldrich, Milan, Italy, cat. L-1516). Subsequently, sections were incubated for 1 h at RT with streptavidin Texas Red (1:1000, Vector Labs, Burlingame, CA, USA). After several PBS rinses, the sections were mounted on gelatin-coated glass slides and observed with a confocal microscope.

For the detection of PSD-95 and gephyrin immunoreactivity, mice were anesthetized intraperitoneally using Avertin (Sigma-Aldrich) and decapitated. The brains were rapidly excised and cut into coronal slabs that were fixed by immersion in ice-cold 4% formaldehyde in 0.1 M PB for 30 min. After fixation, the tissue slabs were rinsed in PB, cryoprotected via immersion in ascending sucrose solutions (10%, 20%, and 30%), cut into 20 μm sections with a cryostat, mounted on gelatin-coated slides and stored for a maximum of 1 month at −20°C (Giustetto et al., 1998). Following a blocking step in PBS with 10% NDS and 0.5% Triton X-100, the sections were incubated with the primary antibodies (Table 1) overnight at 4°C. The sections were then washed and incubated with Cy3 anti-mouse secondary antibodies (1:1000; Jackson ImmunoResearch, West Grove, PA, USA) for 1 h at room temperature. The sections were then rinsed again and coverslipped with Dako medium.

### Image Analysis

All analyses were carried out in the binocular region of V1, which was identified according to anatomical marks (van Brussel et al., 2009), by an investigator who was blinded to the genotype. Cortical layers were identified as in Tomassy et al. (2014).

The analysis of c-Fos^+^ cell density was performed under constant microscope settings and bright-field illumination intensity. Images were background subtracted with ImageJ using the intensity of corpus callosum background staining as threshold, and cells were manually counted using the point tool in ImageJ.

Synaptic puncta in the neuropil were analyzed from 5 to 6 mice per genotype on five serial optical sections (0.5 μm Z-step size) that were acquired from layers 2 to 5 of the V1 cortex with a laser scanning confocal microscope (LSM5 Pascal; Zeiss, DE, Germany) using a 100× objective (1.4 numerical aperture) and the pinhole set at 1 Airy unit. Synaptic puncta were identified if present in at least two consecutive optical sections. PV^+^-VGAT^+^ puncta that outlined the cell body of pyramidal neurons and VGluT1^+^ puncta in close juxtaposition to a labeled dendritic segment were identified as presynaptic varicosities. The co-apposition of VGluT1^+^ clusters with PV/CR-positive dendrites was estimated visually in the three orthogonal planes with an Imaris Software dedicated tool. The number of immunopositive puncta was determined by manually counting pre- or post-synaptic clusters using the Imaris Software (Bitplane, Switzerland) and expressed as puncta/μm^2^ (neuropil density) or puncta/μm (somata/dendritic density).

The density of INs and WFA^+^ PNNs was estimated in at least five animals per genotype. Confocal images spanning from the pial surface to the white matter of V1 were captured in at least two corresponding coronal brain sections with a 40× objective (1.0 NA) using a 1-μm Z-step. Digital boxes in width (230.34 μm) spanning from the pial surface to the white matter (corpus callosum) were superimposed at matched locations on each coronal section of the V1 cortex, divided into 10 equally sized sampling areas (bins; layer I: bin 1; layer II/III: bins 2–3; layer IV: bins 4–5; layer V: bins 6–7; layer VI: bins 8–10), and immunopositive cells were manually counted in each bin (Tomassy et al., 2014).

The analysis of WFA staining intensity was performed as previously described (Foscarin et al., 2011) on confocal images captured with a 100× objective (at least 30 PNNs/animal and five animals per genotype). To minimize intensity variability, each labeled PNN was acquired in a single confocal section showing the highest WFA intensity. Briefly, the brightness intensity (range 0–255) of PNNs was measured by averaging the intensity of 15 pixels randomly selected from each net. The background brightness, taken from a non-stained region of layer I, was subtracted from the brightness measurements. Depending on its mean intensity, each net was included in one of three categories based on the percentage of the maximum staining intensity: faint = 0–33%, medium = 34–66%, strong = 67–100%.

### Statistical Analysis

If the data sets passed normality test and equal variance test, we performed Student’s *t*-test or two-way ANOVA followed by Bonferroni *post hoc* tests as specifically indicated in the text of each figure legend, using GraphPad Prism software (La Jolla, CA, USA). To compare the distribution of frequencies relative to WFA staining intensity categories, we used *χ*^2^-test. All values are presented as mean ± SEM, and *n* indicates the number of mice.

## Results

### Neuronal Activity Is Reduced in V1 of CDKL5^−/y^ Mice

**Figure 1:**
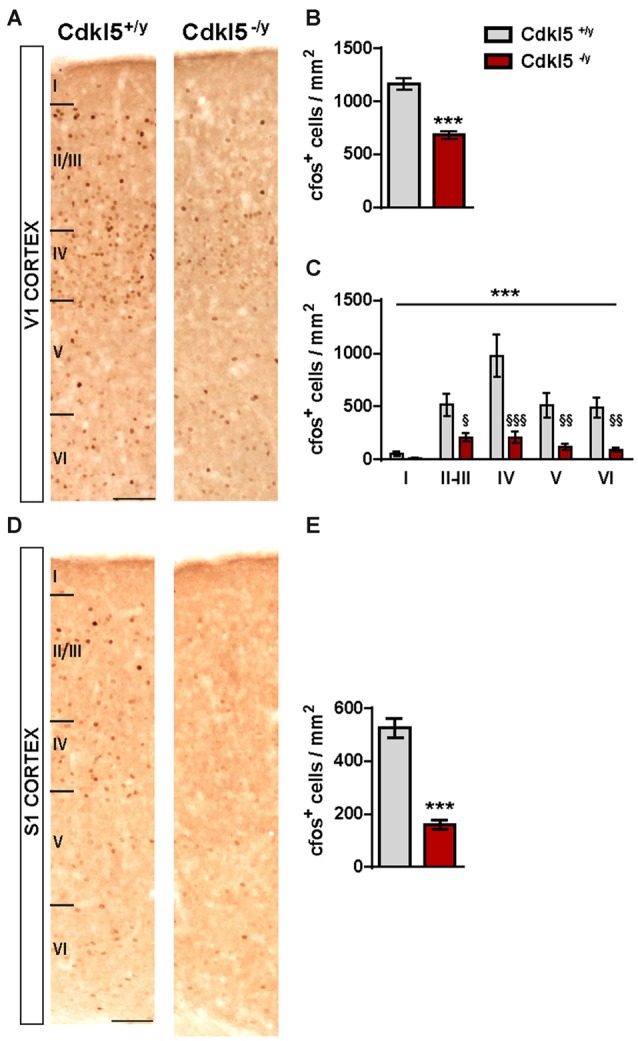
**Neural activity in primary visual cortex (V1) and S1 cortices of adult cyclin-dependent kinase-like 5 (Cdkl5^−/y^) mice is reduced. (A)** Representative examples of c-Fos immunohistochemistry in V1 of Cdkl5^+/y^ and Cdkl5^−/y^ mice (scale bar 50 μm). Analyses of c-Fos^+^ cells density **(B)** and c-Fos cells density layer distribution **(C)** reveal a decrease in c-Fos levels spanning across all cortical layers in mutant mice as compared to WT. **(D)** Representative examples of c-Fos immunohistochemistry in S1 of Cdkl5^+/y^ and Cdkl5^−/y^ mice (scale bar 50 μm) and quantification analysis of c-Fos^+^ cells density **(E)**. c-Fos cell density analysis: Student’s *t*-test, ***p < 0.001; layer distribution: two-way ANOVA, ***p < 0.001; *Post hoc* Bonferroni test, ^§^*p* < 0.05, ^§§^*p* < 0.01, ^§§§^*p* < 0.001.

We evaluated the expression levels of a marker of neuronal activity, the immediate early gene c-Fos, to assess overall levels of cortical activity in 8-weeks old Cdkl5^−/y^ male mice. As shown in Figure 1A, we found that the levels of c-Fos immunoreactivity were strongly reduced throughout V1 layers in mutant mice. Quantitative analyses revealed a profound reduction of c-Fos positive cell density (Cdkl5^+/y^, 1167 ± 56.3 cells/mm^2^; Cdkl5^−/y^, 683.1 ± 39.2; *p* < 0.05; *n* = 6 for each group) in male mutants compared with WT littermates (Figure 1B). Interestingly, the reduction of c-Fos^+^ cells was detectable across all cortical layers with the exception of layer I, which however has a low cellularity (two-way ANOVA: *p* < 0.001; Bonferroni: layer II–III *p* < 0.05, IV *p* < 0.001, V *p* < 0.01, VI *p* < 0.01; Figure 1C). A similar reduction of c-Fos immunolabeling was found in the primary somatosensory cortex (c-Fos^+^ cells density: Cdkl5^+/y^, 525.1 ± 34.84 cells/mm^2^; Cdkl5^−/y^, 159.5 ± 16.37 cells/mm^2^; *p* < 0.001; *n* = 4 for each group; Figures 1D,E) of mutant mice, suggesting that the reduction is generalized in the cortex and not restricted to V1.

### Altered Synaptic Connectivity in V1 of CDKL5^−/y^ Mice

**Figure 2:**
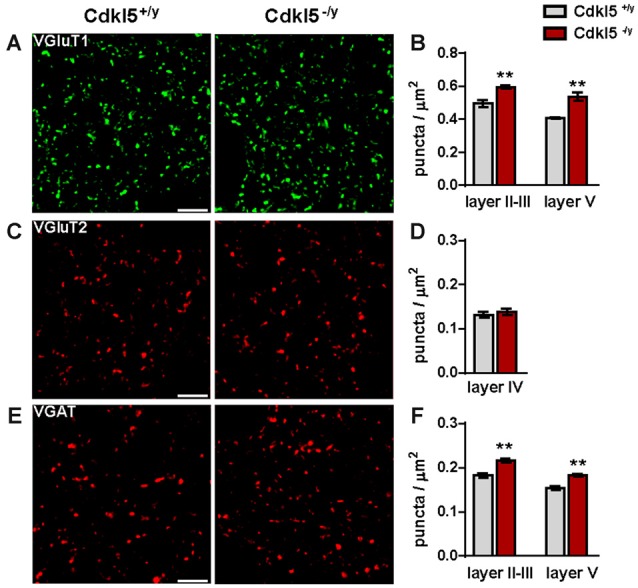
**Synaptic connectivity is altered in V1 of adult Cdkl5^−/y^ mice.** Representative micrographs acquired in the neuropil of V1 illustrating VGluT1^+^ (layer V) **(A)**, VGluT2^+^ (layer IV) **(C)** and VGAT^+^ (layer V) **(E)** immunofluorescence from Cdkl5^+/y^ and Cdkl5^−/y^ mice (scale bar 5 μm). **(B,D,F)** Quantitative analysis of VGluT1^+^ **(B)**, and VGAT^+^ **(F)** puncta shows an increase of both excitatory and inhibitory terminals density in layer II–III and layer V of Cdkl5^−/y^ mice compared to WT whereas no changes were detected in VGluT2^+^ density **(D)**. Puncta density analysis: student’s *t*-test, **p < 0.01.

Because reduced activation of V1 in the absence of CDKL5 may stem from defective neuronal connectivity, we next investigated synaptic organization in V1 using immunofluorescence and confocal microscopy. We first analyzed the localization of the vesicular glutamate transporters 1 (VGluT1) and 2 (VGluT2), which are markers of cortico-cortical and thalamo-cortical glutamatergic axon terminals, respectively (Wojcik et al., 2004; Nahmani and Erisir, 2005), and the vesicular GABA transporter (VGAT), a marker of GABAergic axonal terminals (Chaudhry et al., 1998). Surprisingly, we found a significant increase in the density of VGluT1-positive puncta both in the upper and lower layers of V1 in sections from mutant mice compared to WT littermates (VGluT1^+^ puncta/μm^2^, layer II–III: Cdkl5^+/y^ 0.49 ± 0.02, Cdkl5^−/y^ 0.59 ± 0.01 *p* < 0.01; layer V: Cdkl5^+/y^ 0.41 ± 0.01, Cdkl5^−/y^ 0.54 ± 0.02 *p* < 0.01; *n* = 5–6 for genotype; Figures 2A,B). On the other hand, we detected no changes in the density of VGluT2^+^ puncta in layer IV, where the majority of VGluT2^+^ terminals are located, between WT and KO mice (VGluT2^+^ puncta/μm^2^: Cdkl5^+/y^ 0.13 ± 0.01, Cdkl5^−/y^ 0.14 ± 0.01 *p* = 0.53; *n* = 5–6 for genotype; Figures 2C,D). These findings suggest that thalamo-cortical afferents are preserved in the mutant mice and that lack of CDKL5 has a specific impact on cortico-cortical excitatory synapses.

**Figure 3:**
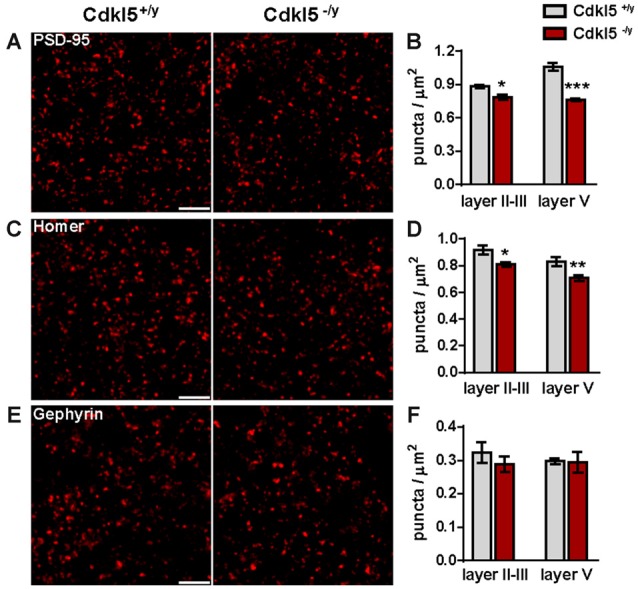
**The molecular organization of excitatory postsynapses is disrupted in Cdkl5^−/y^ mice.** Representative micrographs of PSD-95 **(A)**, Homer **(C)** and gephyrin **(E)** punctuate immunofluorescence in the neuropil (layer V) of V1 from Cdkl5^+/y^ and Cdkl5^−/y^ mice (scale bar 5 μm). Quantitative analysis of immunoreactive puncta reveals a significant reduction of excitatory molecules PSD-95 **(B)**, and Homer **(D)** in layer II–III and V in Cdkl5^−/y^ mice with no changes in gephyrin^+^ puncta **(F)**. Puncta density analysis: Student’s *t*-test, *p < 0.05, **p < 0.01, ***p < 0.001.

Besides, the density of VGAT-immunopositive terminals was also augmented in mutant V1 (VGAT^+^ puncta/μm^2^, layer II–III: Cdkl5^+/y^ 0.18 ± 0.001, Cdkl5^−/y^ 0.22 ± 0.001 *p* < 0.01; layer V: Cdkl5^+/y^ 0.15 ± 0.001, Cdkl5^−/y^ 0.18 ± 0.01 *p* < 0.01; *n* = 5–6 for genotype; Figures 2E,F). This concomitant increase of VGluT1^+^ and VGAT^+^ terminals is apparently in conflict with the reduced activity revealed by c-Fos imaging. To get further insights into the synaptic determinants of altered cortical activity, we next investigated postsynaptic compartments. First, we analyzed the expression of PSD-95, a major scaffolding molecule at the glutamatergic post synaptic site, crucially involved with the control of E/I ratio (Prange et al., 2004; Keith and El-Husseini, 2008). As shown in Figure 3A, the number of PSD-95 immunofluorescent puncta was reduced in V1 of mutant mice compared with control animals (PSD-95^+^ puncta/μm^2^, layer II–III: Cdkl5^+/y^ 0.88 ± 0.01 vs. 0.78 ± 0.02 in Cdkl5^−/y^, *p* < 0.05; layer V: 1.06 ± 0.03 in Cdkl5^+/y^ vs. 0.76 ± 0.01 in Cdkl5^−/y^; *p* < 0.0001; *n* = 5–6 for genotype; Figures 3A,B). Accordingly, we also found a significant decrease in the density of Homer, another postsynaptic constituent that is coupled to PSD-95 by the scaffolding protein Shank (Luo et al., 2012) (Homer^+^ puncta/μm^2^, layer II–III: 0.92 ± 0.03 in Cdkl5^+/y^ vs. 0.81 ± 0.02 in Cdkl5^−/y^, *p* < 0.05; layer V: 0.86 ± 0.03 in Cdkl5^+/y^ vs. 0.71 ± 0.02 in Cdkl5^−/y^, *p* < 0.01; *n* = 5 for genotype; Figures 3C,D). Despite these alterations of glutamatergic postsynapses, we did not find any significant changes in the density of gephyrin, a scaffolding protein involved in regulating the maturation and plasticity of GABAergic synapses (Tyagarajan and Fritschy, 2014) (gephyrin^+^ puncta/μm^2^, layer II–III: 0.32 ± 0.03 in Cdkl5^+/y^ vs. 0.29 ± 0.02 in Cdkl5^−/y^, *p* = 0.41; layer V: 0.30 ± 0.01 in Cdkl5^+/y^ vs. 0.29 ± 0.03 in Cdkl5^−/y^, *p* = 0.92; *n* = 5–6 for genotype; Figures 3E,F). These data indicate that ablation of CDKL5 leads to a selective disruption of key molecular constituents of the glutamatergic PSD, and alters the number of pre- and postsynaptic specializations in an unpredictable manner.

### The Density of Parvalbumin-Positive Interneurons Is Abnormal in V1 of CDKL5^−/y^ Mice

**Figure 4:**
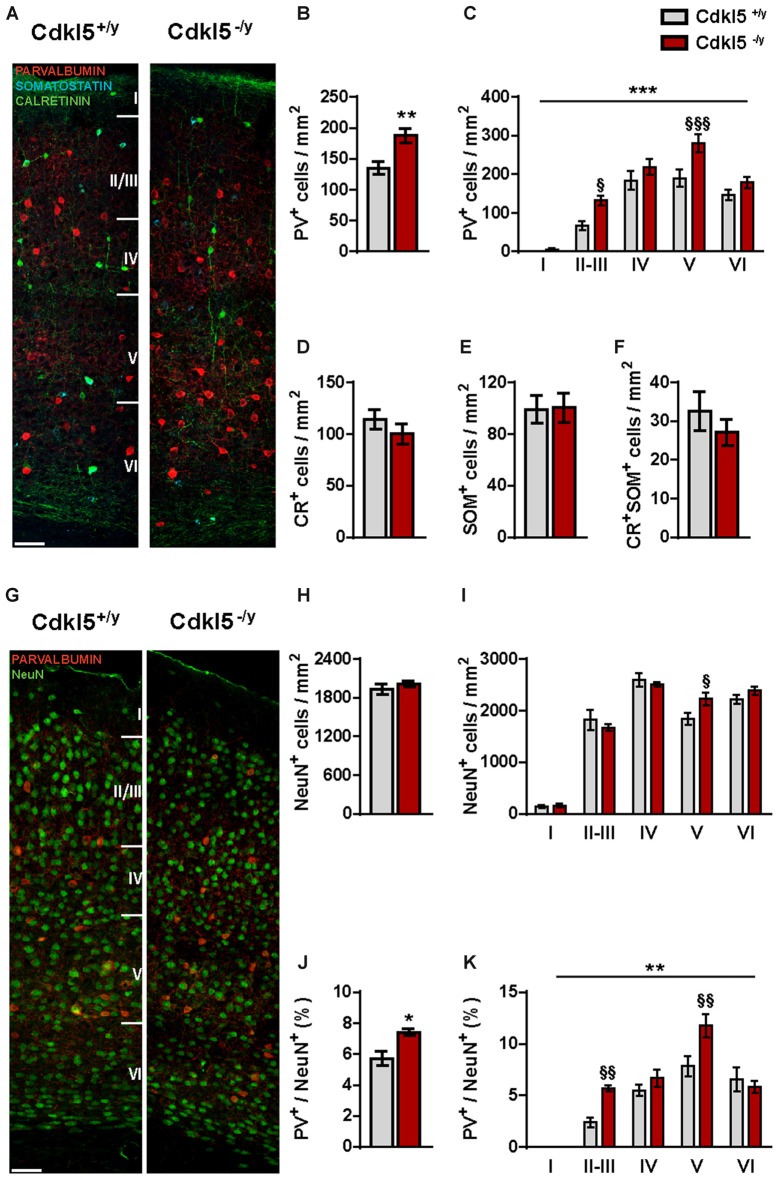
**Cdkl5 deletion affects the organization of neocortical interneurons. (A)** Representative confocal micrographs of triple immunofluorescence for PV^+^ (red), CR^+^ (green) and SOM^+^ (cyan) INs throughout V1 from Cdkl5^+/y^ and Cdkl5^−/y^ mice (scale bar 50 μm). **(B–F)** Quantification of total density **(B)** of INs shows a selective increase of PV^+^ cells in Cdkl5^−/y^ mice compared to WT that is particularly pronounced in layers II–III and V of the cortex **(C)**. The total density of CR^+^ **(D)**, SOM^+^ **(E)** and CR^+^/SOM^+^ **(F)** INs shows no abnormalities. **(G)** Representative confocal micrographs of double immunofluorescence for PV^+^ (red) and NeuN^+^ (green) cells in V1 of Cdkl5^+/y^ and Cdkl5^−/y^ mice (scale bar 50 μm). **(H,I)** Whereas quantification of total NeuN^+^ cells density shows no apparent changes of neuronal density in Cdkl5^−/y^ mice **(H)**, layer distribution analysis reveals a small but selective increase in layer V in mutant mice compared to WT **(I)**. **(J,K)** Total quantification **(J)** and layer distribution **(K)** analyses of PV^+^/NeuN^+^ ratio indicate an increase in the percentage of PV^+^ INs that particularly affects layers II–III and V of Cdkl5^−/y^ mice. Total density analysis: student’s *t*-test, *p < 0.05, **p < 0.01. Layer density analysis: two-way ANOVA, **p < 0.01, ***p < 0.001; *Post hoc* Bonferroni test, ^§^*p* < 0.05, ^§§^*p* < 0.01, ^§§§^*p* < 0.001.

*Cdkl5* is expressed at high levels in inhibitory INs (Rusconi et al., 2008), suggesting that some of the synaptic alterations observed in Cdkl5 KO mice could be associated with altered IN densities. We therefore investigated three major IN subtypes, which account for more than 90% of all cortical INs (Wonders and Anderson, 2006; Gonchar et al., 2007; Fishell and Rudy, 2011; Rudy et al., 2011): the fast-spiking parvalbumin^+^ (PV) INs, the regular-spiking calretinin^+^ (CR) INs, and the burst-spiking somatostatin^+^ (SOM) INs. Moreover, since CR^+^ cells can be further subdivided by the differential expression of SOM (Xu et al., 2006), we also investigated SOM^+^CR^+^ cells. This analysis revealed that the density of PV^+^ cells was significantly increased (134.9 ± 10.28 in Cdkl5^+/y^ vs. 187.5 ± 11.71 in Cdkl5^−/y^, *p* < 0.01 cells/mm^2^; *n* = 10 for genotype) in Cdkl5^−/y^ mice compared to WT littermates (Figures 4A,B). Separate analysis of each cortical layer indicated that the density of PV^+^ cells was particularly increased in layers II–III and V (two-way ANOVA: *p* < 0.001; Bonferroni: layer II–III *p* < 0.05, layer V *p* < 0.001; Figure 4C). In contrast, the number of CR^+^, SOM^+^ and SOM^+^CR^+^ positive cells was similar in both genotypes (CR: 114.3 ± 9, 67 in Cdkl5^+/y^ vs. 100.2 ± 9.65 in Cdkl5^−/y^, *p* = 0.87; SOM: 99.19 ± 10.8 in Cdkl5^+/y^ vs. 100.6 ± 11.28 in Cdkl5^−/y^, *p* = 0.93 cells/mm^2^; CR^+^SOM^+^: 32.60 ± 5.04 in Cdkl5^+/y^ vs. 27.11 ± 3.4 Cdkl5^−/y^, *p* = 0.37 cells/mm^2^; *n* = 5 for genotype; Figures 4D–F). CR^+^ INs can be further subdivided in two functionally distinct subpopulations (Butt et al., 2005; Gelman and Marín, 2010) based on their morphology (bipolar vs. multipolar). Both types had a similar density and distribution in Cdkl5 KO and control mice (Bipolar CR: 23.08 ± 3.35 in Cdkl5^+/y^ vs. 17.75 ± 4.06 in Cdkl5^−/y^, *p* = 0.36; Multipolar CR: 68.68 ± 8.97 in Cdkl5^+/y^ vs. 65.89 ± 7.86 in Cdkl5^−/y^, *p* = 0.82 cells/mm^2^; *n* = 5 for genotype).

Given the increased density of PV^+^ cells, we also evaluated the number of PV^+^ INs relative to the total number of neurons identified with NeuN labelling. As shown in Figures 4G,H, the overall density of NeuN^+^ cells was unaffected by Cdkl5 deletion (Cdkl5^+/y^: 1934 ± 77.82, Cdkl5^−/y^: 2017 ± 43.38, *p* = 0.38 cells/mm^2^; *n* = 5 for genotype). However, the analysis of layer distribution revealed a specific increase of NeuN^+^ cell density in layer V of mutant mice (two-way ANOVA, Bonferroni: layer V *p* < 0.05; Figure 4I). In perfect agreement, the percentage of PV^+^ INs relative to the total NeuN^+^ cells was significantly increased in Cdkl5 mutant mice (5.70% ± 0.46 in Cdkl5^+/y^ vs. 7.40% ± 0.22 in Cdkl5^−/y^, *p* < 0.05; *n* = 5 for genotype; Figure 4J). Layer distribution analysis showed that PV^+^ INs are significantly more abundant in layers II–III and layer V of KO mice (two-way ANOVA: *p* < 0.01; Bonferroni: layer II–III *p* < 0.01, layer V *p* < 0.01; Figure 4K). Overall, these observations indicate that ablation of CDKL5 leads to a selective increase in the density of PV^+^ INs of V1 of adult animals, with no apparent changes in other classes of GABAergic INs.

**Figure 5:**
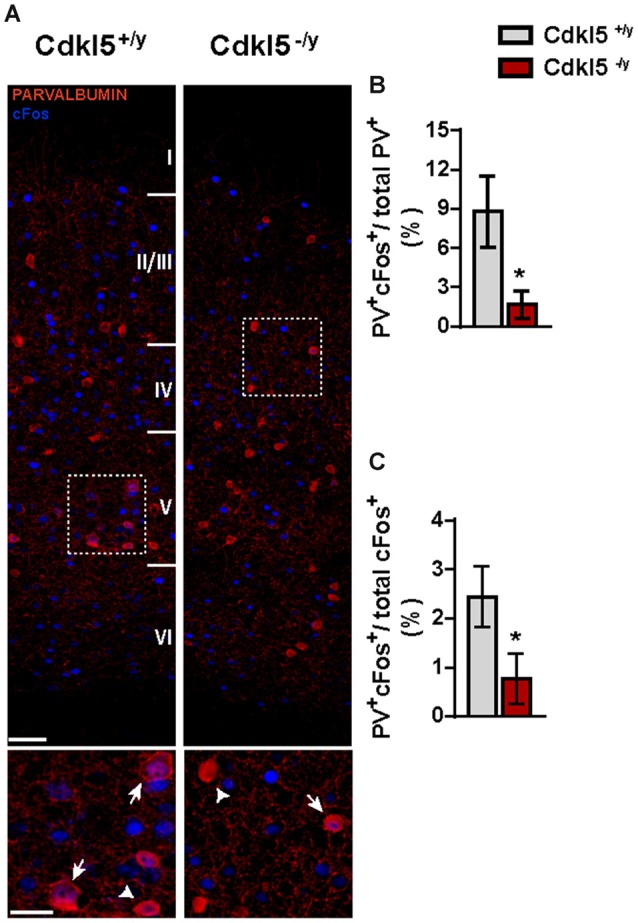
**The number of PV^+^ INs showing c-Fos staining in V1 is reduced in Cdkl5^−/y^ mice. (A)** Representative confocal micrographs (scale bar 50 μm) and higher magnification insets (scale bar 25 μm) showing V1 from Cdkl5^+/y^ and Cdkl5^−/y^ mice stained for PV^+^ (red) and c-Fos^+^ (blue). **(B,C)** Quantitative analyses of c-Fos^+^/PV^+^ INs on total number of PV^+^ INs **(B)** and c-Fos^+^/PV^+^ INs on total number of c-Fos^+^ cells **(C)** reveal a reduction in the percentage of activated PV^+^ INs in mutant mice. Density analysis: student’s *t*-test, *p < 0.05.

Lastly, we verified whether the reduced c-Fos expression observed in V1 of Cdkl5^−/y^ mutants (Figure 1) also affects PV^+^ cells. We found that in WT animals c-Fos was expressed in a small percentage of PV^+^ cells, and this fraction was even smaller in Cdkl5^−/y^ mice (PV^+^c-Fos^+^/total PV^+^: 8.77% ± 2.7 in Cdkl5^+/y^ vs. 1.66% ± 1.06 in Cdkl5^−/y^, *p* < 0.05; *n* = 5 for genotype). Reciprocally, the number of c-Fos^+^ cells also expressing PV was also reduced (PV^+^c-Fos^+^/total c-Fos^+^: 2.44 ± 0.62 in Cdkl5^+/y^ vs. 0.76 ± 0.51 in Cdkl5^−/y^, *p* < 0.05; *n* = 5 for genotype; Figures 5A–C). Thus, c-Fos is normally expressed by a small percentage of PV^+^ INs, and c-Fos expression reveals a small but significant reduction of PV^+^ cell activation in Cdkl5^−/y^ mice.

### Enhanced Connectivity Between Parvalbumin Interneurons and Pyramidal Cells in **CDKL5^−/y^** **Mice**

**Figure 6:**
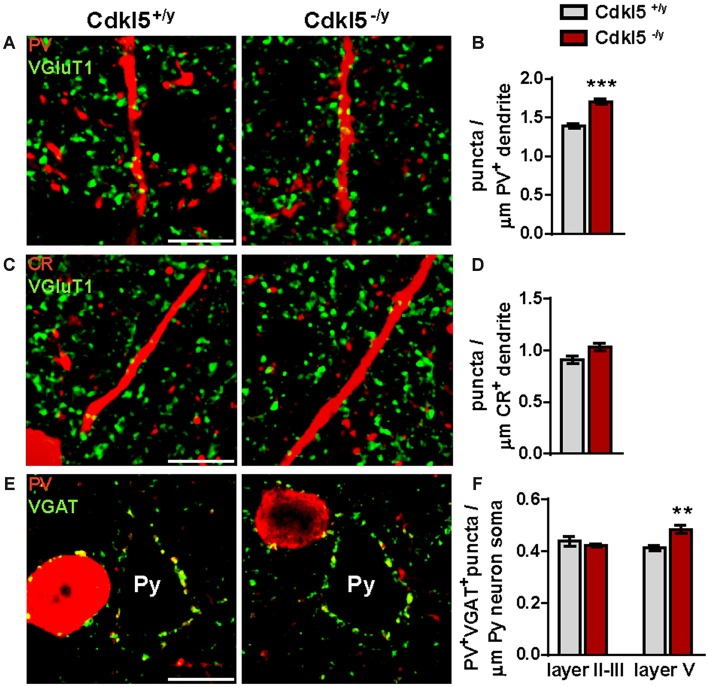
**The connectivity between PV^+^ INs and pyramidal cells is enhanced in V1 of Cdkl5^−/y^ mice. (A,C)** Examples of VGluT1^+^ varicosities in V1 decorating dendritic branches of PV^+^ **(A)** and CR^+^ **(C)** INs from both Cdkl5^+/y^ and Cdkl5^−/y^ mice (scale bar 10 μm). **(B,D)** Analysis of VGluT1^+^ terminals contacting dendrites reveals an increase in excitatory connectivity on PV^+^ INs in Cdkl5^−/y^ mice compared to WT **(B)** while no changes in excitatory terminals were detected on CR^+^ cells **(D)**. **(E)** Representative micrographs of PV^+^/VGAT^+^ puncta decorating the somata of pyramidal neurons in V1 of Cdkl5^+/y^ and Cdkl5^−/y^ mice (scale bar 10 μm). Py, pyramidal neurons. Quantitative analysis **(F)** shows an increase of PV^+^ GABAergic synapses on the somata of layer V pyramidal neurons in mutant mice. Puncta density analysis: student’s *t*-test, **p < 0.01, ***p < 0.001.

Given the abnormal density of PV^+^ subclass of INs in V1 of Cdkl5 KO mice, we decided to investigate whether Cdkl5 deletion may also have an impact on the structural connectivity of these cells. First, we investigated the weight of synaptic input by evaluating the number of excitatory connections onto PV^+^ INs throughout all cortical layers. We found an higher density of excitatory terminals decorating the dendrites of PV^+^ INs in Cdkl5 null animals compared with WT mice (VGluT1^+^ puncta/μm, 1.39 ± 0.03 in Cdkl5^+/y^ vs. 1.70 ± 0.04 in Cdkl5^−/y^, *p* < 0.001; 8 dendrites/animal, *n* = 5 for genotype; Figures 6A,B). In support of a cell-specific action of Cdkl5 on INs organization, we did not find any difference in the number of excitatory inputs contacting the dendrites of CR^+^ INs (VGluT1^+^ puncta/μm, 0.91 ± 0.04 in Cdkl5^+/y^ vs. 1.033 ± 0.04 in Cdkl5^−/y^, *p* = 0.058; 8 dendrites/animal, *n* = 5 for genotype; Figures 6C,D).

We then investigated the synaptic output of PV^+^ cells by counting the number of GABAergic synapses established by these cells with the cell body of pyramidal neurons. This analysis revealed a significant increase of the density of double-labeled (VGAT^+^/PV^+^) boutons decorating the cell body of layer V pyramidal neurons in mutant mice compared to WT animals (PV^+^VGAT^+^ puncta/μm, 0.41 ± 0.01 in Cdkl5^+/y^ vs. 0.49 ± 0.02 in Cdkl5^−/y^, *p* < 0.01; 8 pyramidal cell soma/animal, *n* = 5 for genotype; Figures 6E,F). Surprisingly, no changes were found around the cell body of layer II/III pyramidal neurons (PV^+^VGAT^+^ puncta/μm, 0.41 ± 0.01 in Cdkl5^+/y^ vs. 0.40 ± 0.01 in Cdkl5^−/y^, *p* = 0.69; 8 pyramidal cell soma/animal, *n* = 5 for genotype). Together, these data reveal that the lack of CDKL5 leads to excessive glutamatergic connectivity onto PV^+^ cells in V1 that in turns make more synapses with corticofugal layer V pyramidal neurons.

### The Density of Perineuronal Nets Is Abnormal in V1 Cortex of Adult **CDKL5^−/y^** **Mice**

**Figure 7:**
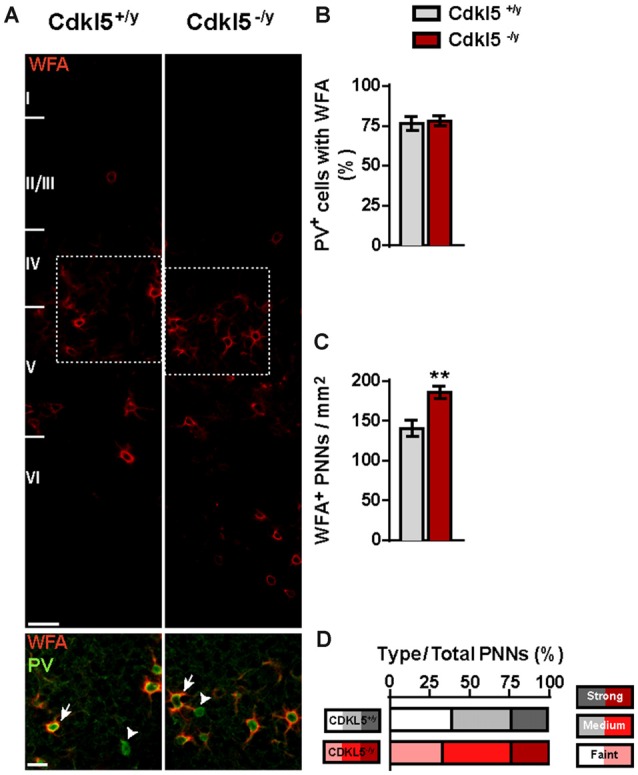
**CDLK5 loss affects peri-neuronal nets (PNNs) density in adulthood. (A)** Representative confocal micrographs of wisteria floribunda agglutinin (WFA)^+^ PNNs in V1 of 8-week old Cdkl5^+/y^ and Cdkl5^−/y^ mice (scale bar 50 μm) and higher magnification insets showing PV^+^ INs (arrowheads) and PV^+^ cells enwrapped by WFA^+^ PNNs (arrows; scale bar 25 μm). **(B,C)** Analysis of the percentage of PV^+^ cells enwrapped by PNNs shows no differences between WT and mutant mice **(B)** whereas the quantification of the total density of PNNs reveals an increase in Cdkl5^−/y^ mice **(C)**. **(D)** The analysis of the frequency distribution of PNNs maturation, based on WFA^+^ staining intensity, does not detect any changes between wild type and mutant mice. PNNs density analysis: student’s *t*-test, **p < 0.01.

To further characterize the effects of Cdkl5 deletion on PV^+^ cells, we next evaluated the density and pattern of expression of chondroitin sulfate proteoglycans (CSPGs) visualized with WFA labeling. CSPGs condense around the cell body and major dendrites of PV^+^ INs forming extracellular matrix specialized structures called PNNs (Wang and Fawcett, 2012). PNNs were reported to progressively limit the plasticity of PV^+^ INs with the establishment of mature circuits (Pizzorusso et al., 2002; Kwok et al., 2011). As shown in Figure 7B, the percentage of PV^+^ cells enwrapped by PNNs was similar in both genotypes (76.39% ± 4.43 in Cdkl5^+/y^ vs. 78.15% ± 3.12 in Cdkl5^−/y^, *p* = 0.76; *n* = 5 for genotype). However, the density of cells surrounded by WFA^+^PNNs in V1 of mutant mice showed an increase compared to WT (140.1 ± 10.19 in Cdkl5^+/y^ vs. 185.7 ± 8.04 in Cdkl5^−/y^, *p* < 0.01; *n* = 5 for genotype), consistent with the augmented density of PV^+^ INs (Figures 7A,C). Despite this, there were no obvious changes in the intensity of WFA^+^ PNNs in Cdkl5^+/y^ and control mice (*χ*^2^ Cdkl5^+/y^ vs. Cdkl5^−/y^, *p* = 0.97; *n* = 5 for genotype; Figure 7D). Thus, the increase of PV^+^ INs in Cdkl5^−/y^ mutants is accompanied by an increase in the number of cells enwrapped by PNNs, although the general organization of PNNs is similar in both genotypes.

### Cdkl5 Ablation Affects the Organization of the PV^+^ System During the Critical Period

**Figure 8:**
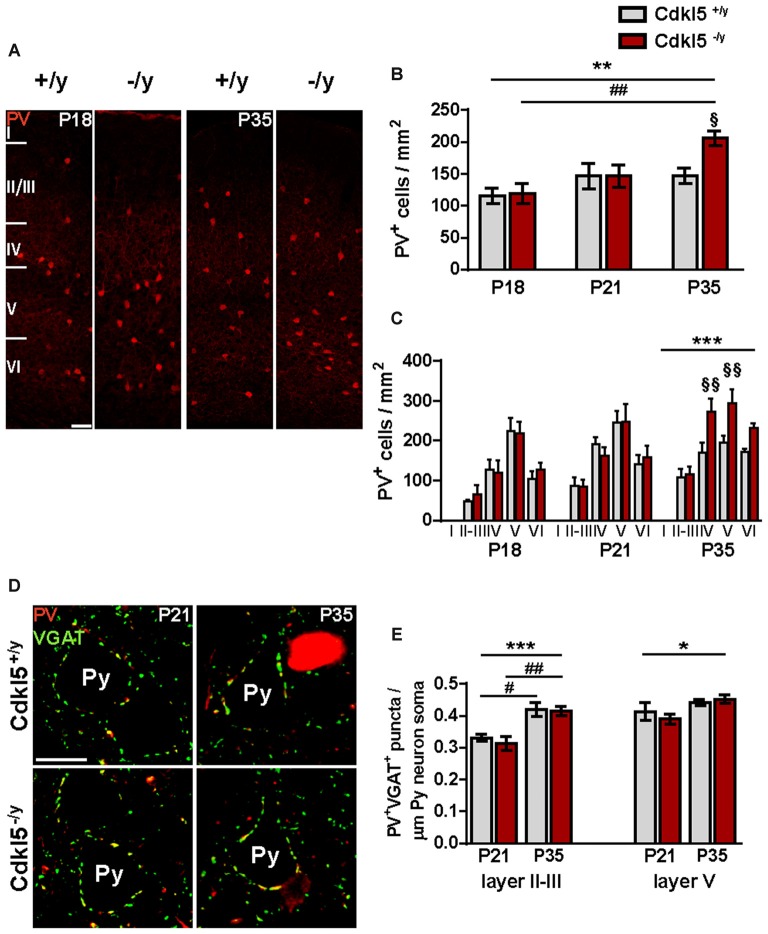
**CDLK5 loss affects PV^+^ INs density during the critical period (CP) of cortical plasticity. (A)** Representative confocal micrographs of PV^+^ INs in V1 of Cdkl5^+/y^ and Cdkl5^−/y^ mice before the onset (P18) and at the end (P35) of the CP (scale bar 50 μm). **(B,C)** Quantitative analyses of total PV^+^ cells density **(B)** and layer distribution **(C)** at P18, P21 and P35 show an age-dependent increase of PV^+^ INs which is abnormally enhanced in mutant mice. **(D)** Examples of PV^+^/VGAT^+^ puncta contacting the somata of layer V pyramidal neurons in in V1 from Cdkl5^+/y^ and Cdkl5^−/y^ mice aged P21 and P35 (scale bar 10 μm). Py, pyramidal neurons. Quantitative analysis **(E)** shows no changes in the density of PV^+^ inhibitory varicosities between WT and mutant mice at either development stages. However, there is an age dependent increase of PV^+^ putative synapses irrespective of genotype. PV^+^ INs density and PV^+^/VGAT^+^ puncta analyses: two-way ANOVA, *p < 0.05, **p < 0.01, ***p < 0.001; *Post hoc* Bonferroni test: ^§^*p* < 0.05, ^§§^*p* < 0.01 compared to the corresponding age-matched wild type group; ^#^*p* < 0.05, ^##^*p* < 0.01 compared to the corresponding genotype-matched P18/21 group.

Because the correct organization of the PV system is crucial for the CP of ocular dominance plasticity (ODP) in the visual cortex (Levelt and Hübener, 2012), we investigated whether Cdkl5 deletion may tamper with the developmental trajectory of cortical circuitry refinement. First, we analyzed the density of PV^+^ INs in a pre-CP stage (postnatal day P18), at the transition toward the opening of the CP (postnatal day P21), and at the end of the CP (postnatal day P35) (Fagiolini et al., 2004; Hooks and Chen, 2007; Sugiyama et al., 2008; Levelt and Hübener, 2012; Sun et al., 2016). While the density of PV^+^ cells was similar in WT and mutant mice at P18 and P21 (two-way ANOVA: *p* > 0.05), null mice displayed a higher number of PV^+^ INs at the closure of the CP (two-way ANOVA: *p* < 0.01; Bonferroni: P35 Cdkl5^+/y^ vs. Cdkl5^−/y^ *p* < 0.05; *n* = 5 for group; Figures 8A,B). Interestingly, the analysis of layer distribution pointed out a selective increase of PV^+^ cells in layers IV–V of mutant mice (two-way ANOVA: *p* < 0.001; Bonferroni: P35 layer IV Cdkl5^+/y^ vs. Cdkl5^−/ y^ *p* < 0.01; layer V Cdkl5^+/y^ vs. Cdkl5^−/y^ *p* < 0.01; Figure 8C). Furthermore, this analysis showed an age-dependent increase of PV^+^ INs in KO mice that was not detected in WT animals (Bonferroni: Cdkl5^−/y^ at P18 vs. Cdkl5^−/y^ at P35 *p* < 0.01; Figure 8B). Intriguingly, our data show that while in adulthood the increase in PV^+^ cell density is detectable in layers II–III and V, at P35 the increase is limited to layers IV–V of mutant mice.

Given the increased density of PV^+^ cells at the end of the CP, we investigated possible abnormalities of their connectivity. Specifically, we quantified the density of PV^+^ terminals targeting the cell body of pyramidal cells at P21 and P35. This analysis revealed no genotype-related abnormalities at both developmental stages (two-way ANOVA: *p* > 0.05; Figures 8D,E). However, in agreement with previous findings (Huang et al., 1999), we observed an age-dependent increase in the density of inhibitory perisomatic synapses in both genotypes (layer II–III, two-way ANOVA: *p* < 0.001; Bonferroni: Cdkl5^+/y^ at P21 vs. Cdkl5^+/y^ at P35 *p* < 0.05; Cdkl5^−/y^ at P21 vs. Cdkl5^−/y^ at P35 *p* < 0.01. layer V, two-way ANOVA: *p* < 0.05; *n* = 4 for group).

**Figure 9:**
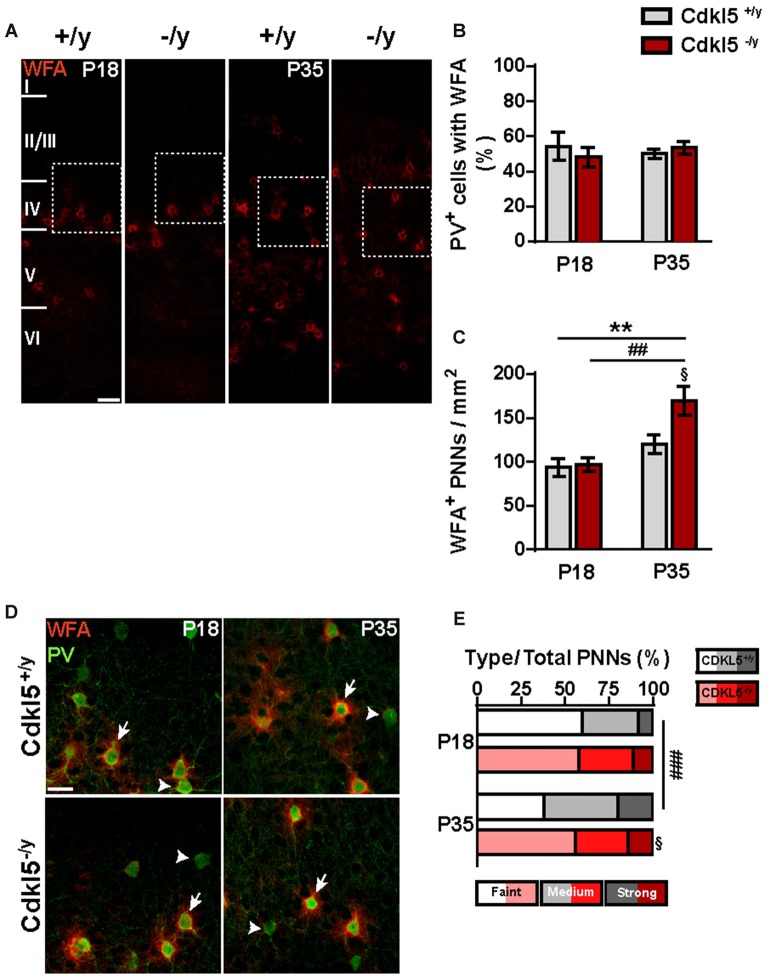
**CDLK5 deletion slows down PNNs organization during the CP of cortical plasticity. (A)** Examples of WFA^+^ PNNs staining in wild type and mutant mice at P18 and P35 (scale bar 50 μm). **(B,C)** Quantification of the percentage of PV^+^ INs surrounded by WFA^+^ PNNs reveals no differences between Cdkl5^+/y^ and Cdkl5^−/y^ mice at either developmental stages **(B)**. The density of WFA^+^ PNNs shows a significant age-dependent increase which is more pronounced in the mutant mice **(C)**. **(D)** Higher magnification insets showing PV^+^ INs (arrowheads) and PV^+^ cells enwrapped by WFA^+^ PNNs (arrows; scale bar 25 μm). **(E)** The analysis of the frequency distribution of PNNs, classified on the basis of their WFA^+^ staining intensity, revealed an age-dependent increase only in wild type mice. PNNs density analysis: two-way ANOVA, **p < 0.01, *Post hoc* Bonferroni test: ^##^*p* < 0.01 compared to the corresponding genotype-matched P18 group, ^§^*p* < 0.05 compared to the corresponding age-matched wild type group. Frequency distribution analysis: *χ*^2^, ^###^*p* < 0.001 compared to the corresponding genotype-matched P18 group, ^§^*p* < 0.05 compared to the corresponding age-matched wild type group.

Since the mature configuration of PNNs coincides with the end of the CP in V1 (Kwok et al., 2011), we evaluated the numerical density and staining intensity of WFA^+^ PNNs before the opening and at the end of the CP. Interestingly, we found no difference in the percentage of PV^+^ INs surrounded by PNNs irrespective of the developmental stage and genotype (two-way ANOVA: *p* > 0.05; *n* = 5 for group; Figures 9A,B). The density and labeling intensity of WFA-positive nets were similar in Cdkl5 KO and control mice at P18 (*χ*^2^ Cdkl5^+/y^ vs. Cdkl5^−/y^, *p* = 0.77; *n* = 5 for group; Figures 9D,E). However, at P35 PNNs were more numerous in Cdkl5 KOs compared with WT mice (two-way ANOVA: *p* < 0.01; Bonferroni: P35 Cdkl5^+/y^ vs. Cdkl5^−/y^ *p* < 0.05, Cdkl5^−/y^ at P18 vs. Cdkl5^−/y^ at P35 *p* < 0.01; *n* = 5 for group; Figure 9C), and WFA staining was significantly fainter in mutants (*χ*^2^ Cdkl5^+/y^ vs. Cdkl5^−/y^, *p* < 0.05; *n* = 5 for group; Figure 9E). Importantly, while PNNs showed an age-dependent process of maturation in WT animals, reflected by an increased frequency of PNNs with strong WFA staining (*χ*^2^ Cdkl5^+/y^ at P18 vs. Cdkl5^+/y^ at P35, *p* < 0.001; *n* = 5 for group), such developmental maturation of PNNs was completely abolished in Cdkl5^−/y^ mice (*χ*^2^ Cdkl5^−/y^ at P18 vs. Cdkl5^−/y^ at P35, *p* > 0.05; *n* = 5 for group). These data indicate that the lack of CDKL5 leads to profound alterations in the establishment of PV^+^ IN assembly, together with defective formation of PNNs at the end of the CP.

## Discussion

In the present study, we report that ablation of CDKL5 profoundly affects synaptic connectivity in the V1. We show that both excitatory and inhibitory presynaptic terminals are increased in V1 of Cdkl5 KO mice. Despite this, we reveal a downregulation of glutamatergic postsynaptic proteins, such as PSD-95 and Homer, with no apparent changes in the density of gephyrin. Furthermore, we demonstrate a selective increase in the number of PV^+^ INs, and of their synapses with pyramidal cells. These defects are functionally relevant, as pointed out by the reduced expression of the neural activity marker c-Fos in V1 of Cdkl5 KO mice. Our data show that lack of CDKL5 has a negative impact on PNN maturation in V1, suggesting that this gene may play a role in mechanisms underlying the closure of the CP. Overall, our data indicate that CDKL5 deletion produces an opposite impact on excitatory and inhibitory transmission in cortical circuits, with an increase in PV^+^-mediated inhibition and a perturbation of glutamatergic synapses, conceivably resulting in alteration of the E/I balance. Furthermore, we provide an anatomical substrate for the visual deficits affecting both CDKL5 patients and the KO mouse model by disclosing developmental alterations that are crucially involved in experience-dependent cortical plasticity.

One important finding of this study is that loss of CDKL5 caused a disruption of the postsynaptic machinery at glutamatergic synapses. Both PSD-95 and Homer clusters were significantly reduced in cortical layers of Cdkl5 KO mice compared with the control littermates. This is consistent with previous findings showing that CDKL5 plays a critical role in orchestrating the molecular composition, stabilization and functionality of excitatory postsynapses and that its absence severely affects the morphology and dynamics of dendritic spines, possibly destabilizing PSD-95-NGL-1 interaction (Ricciardi et al., 2012; Della Sala et al., 2016). Intriguingly, alteration of excitatory transmission was reported recently by studies conducted on neurons derived from CDKL5 mutated human iPS cells (Ricciardi et al., 2012). In particular, these investigations revealed a profound reduction in the expression of excitatory markers such as VGluT1 and PSD-95, combined with an increased presence of filopodia-like immature spines, in CDKL5-derived IPS cells.

Unexpectedly, in our mouse model the reduced expression of PSD-95 was accompanied by an increased number of putative excitatory terminals labeled for VGluT1. This observation was surprising, because two previous studies reported that both PSD-95 and VGluT1 levels are reduced in CDKL5 deficient mice (Ricciardi et al., 2012; Fuchs et al., 2015). It is worth noting that in Ricciardi et al. (2012) CDKL5 was manipulated by *in utero* electroporation, resulting in a downregulation of CDKL5 protein rather than a complete loss. Moreover, the analysis of VGluT1^+^ synapses was conducted on P11 mouse brains, whereas our present data were obtained in fully-mature adult mice. Since synaptogenesis occurs largely during the first postnatal month (De Felipe et al., 1997), we speculate that the reduction of VGluT1^+^ puncta at P11 may just reflect a temporary delay in synaptogenesis, which may not be conserved into adulthood. This hypothesis is also supported by findings from Chen et al. (2010), showing a postnatal migration delay of pyramidal cortical neurons in rats silenced for CDKL5. In Fuchs et al. (2015), the reduction of VGluT1 was revealed in the molecular layer of the hippocampal dentate gyrus, a brain structure that markedly differs from the visual cortex. It is thus plausible that Cdkl5 deletion may result in region-specific abnormalities of synaptic connectivity. Furthermore, in the aforementioned article, the analysis of VGluT1 was carried out by measuring the optical density of VGluT1 immunoreactivity, which correlates with VGluT1 expression levels rather than with the density of VGluT1^+^ clusters corresponding to pre-synaptic structures. The apparent discrepancy between the increase of VGluT1^+^ presynaptic terminals and the reduction of excitatory postsynaptic proteins could also be attributed to a compensatory mechanism established to counteract reduced postsynaptic functionality and/or it could indicate the occurrence of silent synapses, similar to the situation reported in PSD-95 KO mice (Béïque et al., 2006). In line with this possibility, the surface expression of GluR1 was reduced in CDKL5 shRNA silenced neurons (Zhu et al., 2013).

In addition to changes of glutamatergic synapses, we found an increased number of GABAergic axonal boutons in Cdkl5 mutants. This observation is consistent with the increase in the number of PV^+^ INs in mutant V1 (see below). However, the density of postsynaptic gephyrin clusters was similar in Cdkl5 KO and control mice. One possible explanation for this discrepancy is that in the absence of CDKL5 PV^+^, INs establish supernumerary inhibitory synapses with the cell body of pyramidal neurons, where gephyrin is undetectable (Patrizi et al., 2008). At present, it is impossible to determine whether the augmented density of GABAergic axon terminals is exclusively due to the increase of PV^+^ cells and their connections or whether it reflects a more general rearrangement of GABAergic synapses.

PV^+^ cells were the only type of inhibitory IN modified after ablation of Cdkl5. The density of these cells and their synapses was increased in layers II–III and V of mutant V1, and glutamatergic contacts onto PV^+^ dendrites were also increased. These changes are likely to contribute considerably to the visual deficits reported in Cdkl5 KO mice, because PV^+^ cells are involved in mediating feedback and feedforward inhibition and are crucial for the generation of network oscillations underlying perception (Atallah et al., 2012; Lee et al., 2012). These neurons form numerous synapses onto the cell body of pyramidal cells, making them perfectly suited to detect changes in sensory input, to regulate the spiking, and to synchronize brain regions (Kawaguchi and Kubota, 1997). Importantly, the adjustment of the strength of synapses made by PV^+^ INs is needed for equalizing E/I ratio of pyramidal cells following increased activity (Xue et al., 2014). Moreover, pyramidal neurons targeting PV^+^ dendrites represent critical regulators of PV^+^ cell activity (Kameda et al., 2012), and their excitatory synapses can undergo activity-dependent plastic modifications that may be required for rebalancing dynamic changes in network E/I ratio (Chang et al., 2010; Nissen et al., 2010; Le Roux et al., 2013). The hyperconnectivity of pyramidal neurons with PV^+^ cells observed in V1 of Cdkl5 KOs is, therefore, expected to cause a major perturbation of cortical activity, resulting in hypoactivation of projection neurons. Interestingly, several lines of evidences support the concept that dysfunctions of GABAergic circuits specifically involving PV^+^ cells might play a prominent role in RTT syndrome (Chao et al., 2010; Ure et al., 2016). We previously reported an increased density of PV^+^ INs in the somatosensory cortex of MeCP2 KO mice (Tomassy et al., 2014). Durand et al. (2012) also revealed defects in the organization of the PV^+^ circuitry in the V1 of MeCP2 mutants, albeit with no changes in the density of PV^+^ INs.

The reduced expression of c-Fos in V1 of Cdkl5 KO mice (Figure 1) is a strong indicator of an altered E/I balance in cortical circuits. Our observations indicate that c-Fos is expressed in only in a small subset of PV^+^ INs, as reported in previous studies (Mainardi et al., 2009). Interestingly, this small subset of c-Fos^+^ expressing PV^+^ cells is further reduced in the KO mice, suggesting that, hypoactivation of these INs might be a compensatory mechanism to counteract the increased GABAergic drive and/or the strong impairment of glutamatergic transmission. Remarkably, c-Fos levels were also diminished in somatosensory cortex (Figures 1D,E), suggesting that ablation of CDKL5 may cause a generalized reduction of brain circuit activity. A similar hypoactivation of cortical circuits, as revealed by c-Fos expression, was reported in Mecp2 null mice (Kron et al., 2012), indicating that defects in the E/I balance might be a prominent feature of RTT syndrome. Importantly, several lines of evidences suggest that the synaptic and circuitry abnormalities underlying E/I defects in the Mecp2 mice might be region specific. For instance, in the medial prefrontal cortex of Mecp2 null mice, c-Fos reduction is eventually due to reduced excitatory synaptic connectivity with no apparent changes in inhibitory synaptic currents or the expression of PV^+^ (Sceniak et al., 2016), whereas the V1 hypoactivation is caused by enhanced strength of PV^+^ inhibitory connectivity (Durand et al., 2012). This raises the possibility that CDKL5 deletion also has variable effects on the functionality of specific brain circuitries, that may explain the plethora of cognitive, motor and autonomic dysfunctions affecting CDKL5 patients. On the other hand, although early onset seizures are a prominent feature of CDKL5 disorder, surprisingly Cdkl5 KO mice do not exhibit spontaneous seizures, epileptiform activity or increased seizure susceptibility (Wang et al., 2012; Amendola et al., 2014). Thus, it is also conceivable that the overall reduction of cortical activation, that we found in the CDKL5 mouse cortex, might be the consequence of unpredictable compensatory mechanisms responsible for the prevention of seizures onset in the animal.

The mechanisms responsible for the increased density of PV^+^ INs in Cdkl5 mutants are presently unclear. However, CDKL5 was reported to be expressed at high levels in GABAergic INs (Rusconi et al., 2008), positing a possible involvement of CDKL5 in embryonic proliferation and/or cortical migration of these inhibitory neurons. Even though the molecular mechanisms through which the lack of CDKL5 leads to a disordered inhibitory transmission need further investigations, it is worth noting that CDKL5 expression peaks at P14, which coincides with the beginning of PV^+^ expression (Gonchar et al., 2007). Moreover, we report here that the upregulation of PV^+^ INs in Cdkl5 KO mice is already detectable at the closure of the CP, suggesting that CDKL5 loss may affect visual stimuli-driven refinement of cortical circuits during postnatal development. In line with this hypothesis, Ueno et al. (2015) revealed an increase of PV^+^ cells in the visual cortex of dark reared mice.

The structure and function of PNNs play a crucial role in physiological and pathological conditions and, conceivably, tools enabling the manipulation of PNNs may have to some extent a potential for the development of new therapies aimed to normalize neuronal plasticity in neurodevelopmental disorders (Carulli et al., 2016). In this study, we show that the developmental-dependent modifications of PNNs structure, evaluated by WFA intensity, are impaired in CDKL5 KO mice. It is conceivable that the reduced levels of PSD-95 immunoreactivity in Cdkl5 KO cortex, that are already detectable at P28 (Della Sala et al., 2016), could affect visual input processing, as reported by the decrease in the amplitude of visual evoked potentials (VEPs) found in adult Cdkl5^−/y^ mice (Amendola et al., 2014). This reduced strength of visual inputs might lead to a delay in external stimuli-induced maturation of PV^+^ cells and PNNs during the CP. This hypothesis is supported by the maturation sequence of PNNs in Cdkl5 KO mice, in which PNNs intensity appears strongly attenuated at the end of CP, but becomes comparable to WT in adulthood. The same altered visual stimuli-driven refinement of cortex might be responsible for the increase in the number of PV^+^ synapses on pyramidal cells, which is observed in adult mice only. According to this interpretation, the excessive PV^+^ cells in the mutant mice would need a longer time to establish their connections onto excitatory cells, resulting in a surplus of PV^+^ synapses detectable only in adult mice.

These data suggest that the CP might be prolonged in Cdkl5 mutants. Accordingly, PSD-95 KO mice exhibit lifelong ODP (Huang et al., 2015). Interestingly, defects of PV^+^ INs disrupting CP mechanisms were found in V1 of MeCP2 null mice (Krishnan et al., 2015), and conditional deletion of MeCP2 in PV^+^ cells completely abolished CP plasticity (He et al., 2014). However, in MeCP2 KO mice, the maturation of PV^+^ circuitry is accelerated, leading to a premature opening and closure of CP of plasticity in V1. Importantly, in MeCP2 mutants the accelerated maturation of the PV^+^ network was not associated with an increase in the density of PV^+^ INs, as we found in Cdkl5 mutant mice, but rather with increased levels of PV expression (Krishnan et al., 2015). Future experiments combining electrophysiological input-output connectivity evaluation of both PV^+^ INs and pyramidal cells in V1 associated with an analysis of the CP using a monocular deprivation paradigm will help defining the impact of Cdkl5 on cortical function and plasticity.

In conclusion, our study points to a primary role of CDKL5 in the correct formation and maintenance of the E/I balance in V1 *in vivo* that is likely to underlie the severe visual impairments associated with CDKL5 pathology. Furthermore, we unveiled an unknown crucial role of CDKL5 in the organization of cortical inhibitory transmission. Future studies are needed to pinpoint the molecular machinery responsible for CDKL5-mediated E/I balance functionality. Moreover, our study further supports the idea that RTT variants may actually share common pathogenic mechanisms, involving disruption of the E/I ratio and an enhancement of GABAergic transmission mediated by PV^+^ INs (Dani et al., 2005; Durand et al., 2012; Tomassy et al., 2014; Mariani et al., 2015; Patriarchi et al., 2016). This kind of information is important as it may help to design a common treatment for different forms of RTT.

## Author Contributions

RP, MS-P and MG conceived and designed the study. RP, EC and AG performed the experiments. RP, AG, MS-P and MG analyzed the data. EA, CG, RP, AG, MS-P and MG provided reagents, materials and analysis tools. RP, MS-P and MG wrote the manuscript.

## Conflict of Interest Statement

The authors declare that the research was conducted in the absence of any commercial or financial relationships that could be construed as a potential conflict of interest.
